# Comparative in vitro evaluation of the antimicrobial activities of povidone-iodine and other commercially available antiseptics against clinically relevant pathogens

**DOI:** 10.3205/dgkh000376

**Published:** 2021-01-26

**Authors:** Eng Lee Tan, Nur Humaira Johari

**Affiliations:** 1 Singapore Polytechnic, Singapore

**Keywords:** antiseptics, antimicrobial resistance, povidone-iodine, PVP-I, in vitro antimicrobial activity

## Abstract

**Aims:** Antiseptics, such as povidone-iodine (PVP-I), play an important role in infection control across a wide range of clinical settings. This study aimed to evaluate the comparative *in vitro* efficacy and rate of onset of action of a range of formulations of PVP-I and other commonly used antiseptics.

**Methods:** The antimicrobial efficacy of a range of antiseptics and antimicrobial agents used for skin, wound, vagina and oral antisepsis was evaluated according to the EU Standards DIN EN1276 and EN14476. The panel of organisms tested included bacterial and fungal pathogens and two enteroviruses (Coxsackievirus A16 [CA16] and Enterovirus 71 [EV71]).

**Results:** All PVP-I products tested were highly efficacious *in vitro* (>99.99% kill rate) against a range of clinically relevant bacterial and fungal pathogens with rapid onset of action (30–60 seconds), at both high and low concentrations. By comparison, the efficacy of other antiseptics tested was generally reduced upon dilution. PVP-I products used in wound and oral care were found to be more effective *in vitro* against CA16 and EV71, and had a faster onset of action than most other agents tested.

**Conclusion:** This study provides valuable insights into the *in vitro* efficacy of a range of commonly used antiseptics and may help inform the selection of appropriate antiseptics by healthcare professionals.

## Zusammenfassung

**Zielsetzung:** Antiseptika wie Povidon-Iod (PVP-I) spielen eine wichtige Rolle in der Infektionskontrolle in einem breiten klinischen Anwendungsbereich. In der Studie sollte die *in vitro*-Wirksamkeit einer Reihe von Formulierungen auf Basis von PVP-I mit anderen häufig verwendeten Antiseptika verglichen werden.

**Methode:** Gemäß DIN EN1276 und EN14476 wurde die antimikrobielle Wirksamkeit einer Reihe von Antiseptika und antimikrobiellen Wirkstoffen, die in Präparaten zur Anwendung auf Haut, Wunden, sowie zur Intim- und Mundpflege eingesetzt werden, geprüft. Das Panel der Prüforganismen umfasste bakterielle und pilzliche Erreger sowie zwei Enteroviren (Coxsackievirus A16 [CA16] und Enterovirus 71 [EV71]).

**Ergebnisse:** Alle getesteten PVP-I-Produkte waren *in vitro* hochwirksam (>99,99% Abtötungsrate) gegen eine Reihe klinisch relevanter bakterieller und pilzlicher Erreger mit schnellem Wirkungseintritt (30–60 s) sowohl bei hohen als auch bei niedrigen Konzentrationen. Im Vergleich dazu war die Wirksamkeit anderer getesteter Antiseptika bei Verdünnung im Allgemeinen geringer. Zur Wund- und Mundhöhlenantiseptik eingesetzte PVP-I-Produkte erwiesen sich *in vitro* als wirksamer gegen CA16 und EV71 und hatten einen schnelleren Wirkungseintritt als die meisten anderen getesteten Mittel.

**Diskussion:** Durch die Kenntnis der *In-vitro*-Wirksamkeit einer Reihe häufig verwendeter Antiseptika wird die Auswahl geeigneter Antiseptika durch medizinisches Fachpersonal erleichtert.

## Introduction

The rapid rise of antimicrobial resistance (AMR), coupled with the dearth of new antibiotics, presents a significant public health challenge. Therefore, there is a need for approaches that can help to minimize microbial load and the spread of infection, while reducing reliance on antibiotics. Antiseptics are important in infection control in a range of therapeutic areas and healthcare settings, such as wound care, burn care and surgical site infections [1], [2], [3]. Antiseptics exert their antimicrobial effect by pleiotropic mechanisms of action, the development of resistance to antiseptics is considered unlikely [4]. The physicochemical properties, spectrum of activity and approved clinical indications should all be taken into account when selecting an appropriate antiseptic for a particular indication [1].

Povidone-iodine (PVP-I; a non-covalent complex of polyvinylpyrrolidone and iodine) is a broad-spectrum antiseptic with demonstrated *in vitro* efficacy against a wide range of organisms, including bacteria, fungi, viruses and protozoa [5], [6], [7], [8], [9], [10], [11]. The *in vitro* activity of PVP-I has also been demonstrated against antibiotic- and antiseptic-resistant bacterial and fungal strains [12], [13], [14], [15], [16], [17], [18], [19], and against microbial biofilms [20], [21], [22]. PVP-I, in a range of concentrations and formulations, has been in use for over 60 years, and clinical applications include antisepsis of skin, wounds, oral cavity, eyes, vagina, and intra-surgical lavage [4], [23], [24], [25], [26], [27], [28], [29], [30]. 

Given the emergent threat associated with AMR, there is an increasing need for antiseptics that are effective against a wide spectrum of pathogens and have a rapid onset of action. Although the *in vitro* activity of PVP-I in solution is well established [7], newer formulations (e.g. PVP-I in a liposomal hydrogel [PVP-ILH]), are now available [31], and it is important to understand their comparative *in vitro* efficacy. The primary objectives of this study were to evaluate the *in vitro* antimicrobial activity and rate of onset of action of a range of concentrations and formulations of PVP-I, and to compare its efficacy with other commercially available antiseptics.

## Methods

### Bacterial and fungal strains

The bacterial (n=13) and fungal reference strains (n=2) included methicillin-resistant *Staphylococcus aureus* (MRSA) ATCC BAA-44, *Escherichia coli* ATCC 25922, *Enterococcus faecium* ATCC 35667, *Streptococcus* *pyogenes* ATCC 19615, *Pseudomonas aeruginosa* ATCC 27853, *Streptococcus mutans* ATCC 25175, *Haemophilus influenzae* ATCC 10211, *Streptococcus pneumoniae* ATCC 49619, *Streptococcus sanguinis* ATCC 10556, *Klebsiella pneumoniae* ATCC BAA-2146, *Streptococcus agalactiae* ATCC 27956, *Staphylococcus epidermidis* ATCC 12228, *Candida albicans* ATCC 10231 and *Candida glabrata* ATCC15126. Strains were grown on Tryptone Soya Agar (TSA) plates at 37ºC under aerobic conditions.

### Viral strains

Two enteroviruses associated with hand foot and mouth disease (HFMD) were evaluated in this study; Coxsackievirus A16 (CA16) and Enterovirus 71 (EV71). Both EV71 and CA16 were cultivated from Rhabdomyosarcoma (RD) cells. 

### Preparation of antiseptic test solutions

Several PVP-I-based products and other antiseptics and antimicrobial agents used in the areas of skin, wound, vagina, and mouth cavity antisepsis were evaluated (Table 1(Tab. 1)). Liquid and soluble products were tested as received to allow high-concentration testing (80%) and diluted 1:4 (20% v/v or w/v) or 1:10 (8% v/v or w/v) in sterile water. Semi-solid products were diluted 1:1 (sterile water) to achieve a stable suspension (test concentration 40% w/v) and 1:10 (8% w/v). Solid products (e.g., lozenges) were dissolved in sterile water to achieve a final concentration of 1 mg ml^–1^.

### Evaluation of antiseptic activity against bacteria and fungi

Antiseptic efficacy was evaluated under clean conditions using the dilution-neutralization method described in the EU Standard DIN EN1276 [32]. Precultures of test organisms were prepared (TSA plates) and incubated at 37ºC overnight. Test suspensions were prepared in a sterile tube (50 ml) via inoculation of a suitable volume of diluent (tryptone sodium chloride buffer; 1.0 g tryptone, 8.5 g NaCl, 1 l H_2_O) to achieve optical density at 600 nm (OD_600_) of 0.150–0.550 (1.5–5×10^8^ colony forming units [cfu] ml^–1^). Test and control procedures were performed in duplicate and carried out in parallel, as described in the European standard [32].

The test suspension, antiseptic test solution and interfering substance (0.03 g l^–1^ bovine albumin) were incubated at 22ºC for 30 seconds (except *E. faecium*, for which the contact time was 60 seconds, due to the known resilience of enterococci in the presence of antiseptics) [33]. 1 ml of test solution was then transferred to a sterile tube containing 8 ml of appropriate neutralizer. After mixing, the tube was incubated at 22ºC for 5 minutes and 1 ml of the validation suspension (dilution of the test suspension to 3×10^2^–1.6×10^3^ c.f.u. ml^–1^) was added. After incubation for a further 30 minutes, 2x1 ml samples were spread onto TSA plates and incubated for 20–24 hours at 37ºC in an incubator supplied with 5% CO_2_. The number of colonies per plate was counted, and the number of survivors per ml in the test suspension after the contact time and the log_10_ reduction relative to the controls were calculated, as described in the European standard [32]. 

In accordance with the European standard, antiseptics were considered to have reached the defined antimicrobial activity threshold if they achieved a greater than 5-log_10_ reduction for bacteria and a greater than 4-log_10_ reduction for fungi, indicating a greater than 99.999% and greater than 99.99% reduction in cell count, respectively.

### Evaluation of antiseptic activity against enteroviruses

Antiviral efficacy and rate of onset of action of a range of antiseptic products used in wound and oral antisepsis were evaluated in duplicate under clean conditions against EV71 and CA16, using the methods described in the EU Standard DIN EN14476 [34]. A monolayer of RD cells was seeded into 96-well microtiter plates containing maintenance medium (MEM buffer containing 2% FCS and 1% Pen-Strep) and incubated overnight at 37ºC in an incubator supplied with 5% CO_2_. 1 ml of the virus suspension and 8 ml of test product solution were added to 1 ml of interfering suspension (0.03 g l^–1^ bovine albumin) and maintained at 0ºC. At each contact time (EV71: 0.5, 5 and 30 minutes; CA16: 0.5, 1, 2, 5 and 30 minutes), 0.5 ml was transferred to 4.5 ml of ice-cold maintenance medium. Serial dilutions were prepared, and 0.1 ml of each dilution added to the pre-prepared microtiter plate containing a confluent RD cell monolayer. The plate was incubated overnight at 37ºC in an incubator supplied with 5% CO_2_. Viral titer (50% tissue culture infective dose; TCID_50_) and log_10_ reduction relative to the controls were calculated as described [34]. 

In accordance with the European standard, antiseptics were considered to have effective antiviral activity if they achieved a greater than 4 lg reduction, indicating a greater than 99.99% reduction in viral titer.

## Results and discussion

There has been renewed interest in the role of antiseptics in infection control and as part of antiseptic stewardship strategies to reduce reliance on antibiotics [1]. Despite the clinical importance of antiseptics, there are relatively few published studies comparing the *in vitro* efficacy of both different antiseptics and different formulations. In this study, products were tested at high and low concentrations and with short contact times to better reflect real-world use.

### Antibacterial and antifungal activity of antiseptics used in wound antisepsis

When the barrier formed by the skin becomes impaired, rapid infiltration of bacterial pathogens can occur. Effective treatment of the resulting skin and soft tissue infections can present a serious clinical challenge. These infections are most commonly caused by Gram-positive pathogens such as *S. aureus*, *S. pyogenes* and enterococci; however, Gram-negatives, including *E. coli*, *P. aeruginosa* and Proteus mirabilis, and fungi, such as *C. auris*, have also been implicated in wound infections [35], [36]. 

The recorded lg reductions in cell counts for several wound care antiseptics against five bacterial pathogens and one fungal pathogen are summarized in Table 2 (Tab. 2). Most PVP-I formulations tested achieved at least a 5 lg or 4 lg reduction of bacteria or fungi, respectively. This corresponded to a greater than 99.99% kill rate; including against pathogens of particular relevance to wound infections such as MRSA, *E. faecium*, *S. pyogenes* and *P. aeruginosa*. Exceptions included high concentrations of PVP-ILH against *C. albicans* and 10% PVP-I ointment against *E. faecium*. These products regained efficacy at low concentrations (8%), suggesting that viscosity of these products or lack of moisture might have affected the results. 

Wound antisepsis is common practice in some parts of Europe [24] and rapid onset of action is an important and desirable property for antiseptics used in clinical practice. The PVP-I products evaluated in this study were fast acting *in vitro* (30 seconds; 60 seconds for *E. faecium*) against pathogens relevant to wound antisepsis. Compared with PVP-I, chloroxylenol and 70% ethanol were effective when undiluted but lost efficacy upon dilution (Table 2 (Tab. 2)). The remaining products showed good efficacy against some organisms but not others, again losing efficacy when diluted (Table 2 (Tab. 2)). This is important because dilution of the active ingredient occurs in real-world antiseptic use (e.g., during washing or by wound exudate).

The results presented here align with previous reports of the *in vitro* efficacy of PVP-I against a range of bacterial pathogens commonly associated with wound infections [21], [37]. Furthermore, PVP-I was shown to be effective, at both high and low concentrations, *in vitro* against biofilms – an important factor for effective wound antisepsis [20], [21].

### Antibacterial and antifungal activity of antiseptics used in oral antisepsis

A diverse range of pathogens cause common oral and oropharyngeal infections, from gingivitis to influenza, with immunocompromised patients particularly at risk from opportunistic bacterial and fungal oral infections [38], [39], [40]. Organisms of relevance to oral health evaluated in this study included MRSA and *P. aeruginosa*, which are sources of opportunistic infection, *S. mutans*, which is associated with tooth decay, and *S. sanguinis*, which is a commensal organism commonly found in oral biofilm and is a marker of oral health [40].

All PVP-I oral products tested demonstrated good *in vitro* efficacy, with most formulations achieving a greater than 99.99% kill rate against almost all pathogens tested (Table 3 (Tab. 3)). Chlorhexidine 0.2% (mouthwash) was also effective at high concentrations against all pathogens tested, but was ineffective against MRSA (in agreement with the publication by Yoneyama et al.) [41], *S. mutans* and *C. albicans* when diluted. Amylmetacresol lozenges were effective against some pathogens (*P. aeruginosa*, *S. sanguinis*, *S. pyogenes* and *H. influenzae*), particularly at high concentrations. Thymol, benzydamine hydrochloride lozenges and saline were, however, ineffective against all pathogens tested. Benzydamine hydrochloride is a non-steroidal anti-inflammatory drug (NSAID); however, the antimicrobial activity of this and other NSAIDs has been reported in the literature [42]. NSAIDs often act in synergy with other antibiotics or require high concentrations and long contact times, which might explain why benzydamine hydrochloride was ineffective under the conditions tested [43], [44], [45].

Overall, these results are in line with previously published reports for oral PVP-I products [38], [39], [40], [41], [46], [47]. For example, a 0.23% solution of PVP-I was reported to exhibit rapid *in vitro* bactericidal and viricidal activity after 15 seconds against *K. pneumoniae*, *S. pneumoniae*, severe acute respiratory syndrome coronavirus (SARS-CoV), Middle East respiratory syndrome coronavirus (MERS-CoV), influenza A and rotavirus [39]. Furthermore, dental floss coated with PVP-I demonstrated rapid bactericidal activity against a range of pathogens associated with dental caries, and prevented biofilm formation [47]. 

### Antibacterial and antifungal activity of antiseptics used in vaginal antisepsis

Vaginal infections are associated with significant morbidity, and if left untreated can lead to development of pelvic inflammatory disease [48]. *Candida* species are the most common cause of vaginal infection [49]; however, aerobic vaginitis is typically caused by pathogens such as *S. agalactiae*, *E. coli*, *S. aureus* and *K. pneumoniae* [50], [51]. 

The majority of PVP-I products again demonstrated good *in vitro* efficacy against all pathogens tested, including the fungal pathogens *C. albicans* and *C. glabrata*, both undiluted and diluted after 30 seconds contact time (Table 4 (Tab. 4)). Exceptions included 7.5% PVP-I (wash) and 10% PVP-I (douche), which were effective against all pathogens at high concentrations but were not effective against *S. agalactiae* at low concentrations. Lactic acid (1% wash) and chlorhexidine digluconate (0.2% wash) were also effective against most pathogens; however, the lactic acid wash did not achieve a greater than 4-log_10_ reduction of *C. glabrata* at low concentrations, and chlorhexidine digluconate was ineffective against MRSA. Triclocarban was effective against *S. agalactiae* at high concentrations only and did not reach the efficacy threshold against any other pathogens tested. 

### Antibacterial and antifungal activity of antiseptics used in skin antisepsis

Skin infections are most commonly caused by streptococcal species, coryneform bacteria and *S. aureus* [52]. *S. epidermidis*, although part of the normal human epithelial flora, is an opportunistic pathogen that is often at the root of nosocomial infections in immunocompromised patients [53]. The efficacy of six antiseptic products against three bacterial pathogens relevant to skin infection was evaluated. All PVP-I skin products and 0.5% chlorhexidine digluconate in 70% ethanol achieved at least a 5 lg or 4 lg reduction of bacteria or fungi, respectively, at both low and high concentrations against all pathogens tested (Table 5 (Tab. 5)). Ethanol (25.66%) was not effective against any of the pathogens tested, and 70% isopropanol and 38.9% ethanol/38.9% isopropanol solutions were only effective at high concentrations. These results align with the study by Reichel et al., which reported that chlorhexidine in alcohol was more effective in suppressing recolonization of the skin by aerobic flora than alcohol alone [54]. The efficacy of PVP-I in ethanol was not evaluated in this study; however, it has previously demonstrated a similar reduction in bacterial cell count to chlorhexidine in ethanol when handwashing [55]. 

### Antiviral activity of wound and oral antiseptics against common hand, foot and mouth disease viruses

HFMD is a common and highly contagious enteroviral infection that mainly affects infants and children. Clinical manifestations include fever, skin eruptions on the hands and feet, and vesicles in the mouth [56]. Two enteroviruses associated with HFMD were evaluated in this study: CA16, which causes self-limiting HFMD, and EV71 which can cause HFMD with neurological complications and fatality [57]. There are currently no effective antiviral drugs or vaccines available, and existing treatments are symptom-based with little efficacy, especially against EV71 [58]. Public health prevention measures, such as effective hand hygiene, are the primary approaches to reduce transmission of HFMD, and improved understanding of the efficacy and spectrum of activity of common antiseptics may help to inform decision-making [59]. 

**Figure 1:**
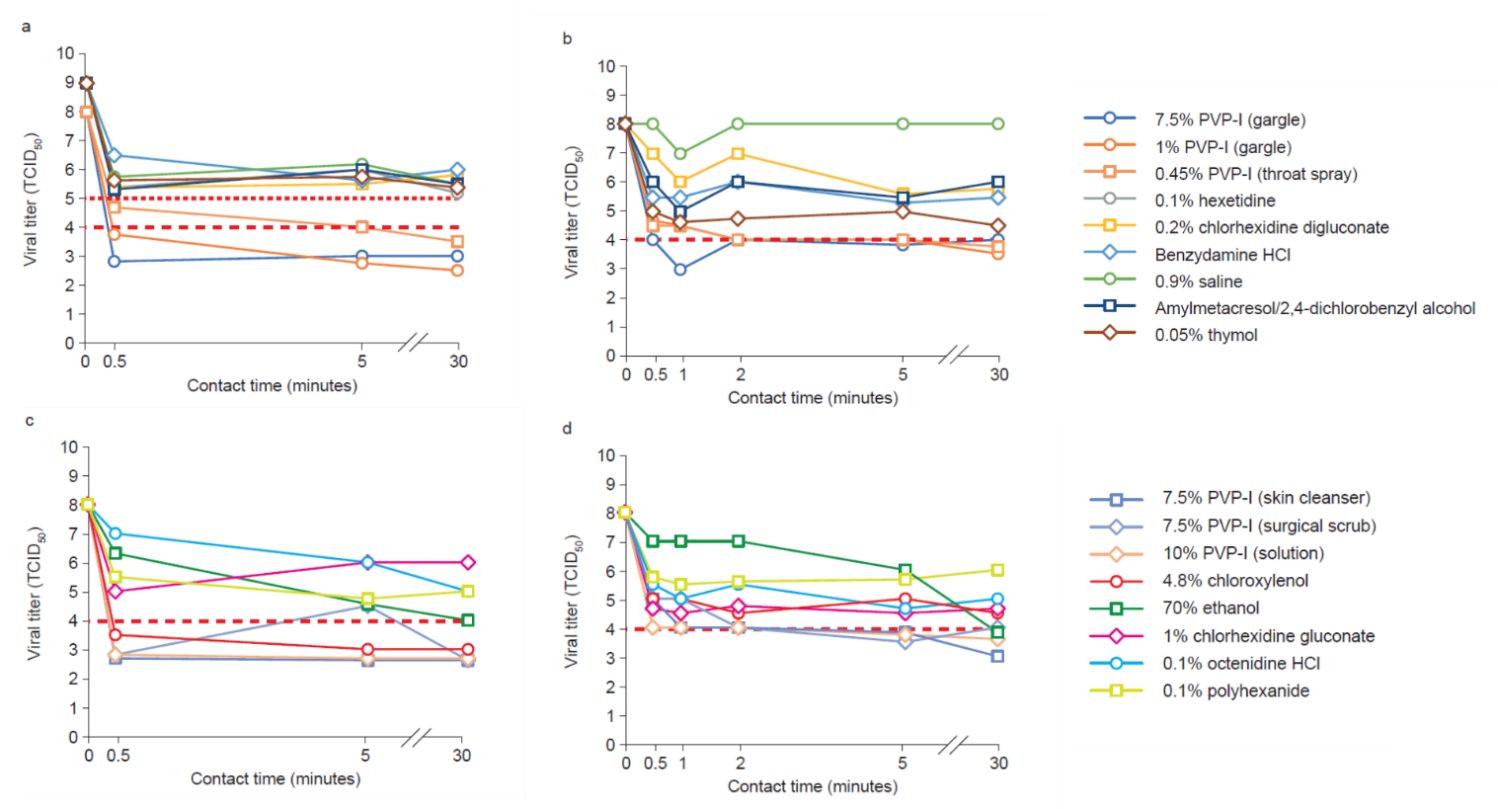
(a) TCID_50_ against EV71 as a function of time for nine oral antiseptic products, (b) TCID_50 _against CA16 as a function of time for eight oral antiseptic products, (c) TCID_50_ against EV71 as a function of contact time for eight wound antiseptic products and (d) TCID_50_ against CA16 as a function of time for eight wound antiseptic products (the red horizontal dotted and dashed lines show the threshold of a 4 lg reduction in viral titer, required to demonstrate antiseptic efficacy as per EU Standard EN14476, for an initial viral titer of 9 and 8, respectively).

The reduction in viral titer for EV71 and CA16 produced by wound and oral antiseptics tested are shown in Figure 1(Fig. 1). Rapid antiviral activity was observed for the oral PVP-I products tested; a greater than 4 lg reduction in viral titer (TCID_50_) was observed between 0.5 and 30 minutes contact time (Figure 1 a, b (Fig. 1)), and the increase in contact time required to reach the efficacy threshold correlated with the decrease in PVP-I concentration. All other oral care antiseptics tested showed weak antiviral activity against both viruses and did not achieve the required efficacy threshold. 

Rapid antiviral activity was also observed for wound care PVP-I products against EV71 and CA16; the required efficacy threshold was reached for all products tested between 0.5 and 2 minutes contact time (Figure 1 c, d (Fig. 1)). Ethanol (70%) showed slower activity against both viruses, achieving a greater than 4 lg reduction after 30 minutes. Chloroxylenol was not effective against CA16, and the remaining wound antiseptics tested showed weak antiviral activity against both viruses. *In vitro* efficacy of PVP-I against SARS-CoV [39], SARS-CoV-2, the causative virus of COVID-19 [60], MERS-CoV [61], rotavirus (strain Wa) [39], influenza A [39], [62], [63], modified vaccinia Ankara (MVA), and Ebola [58] has been reported. The viricidal efficacy of PVP-I observed here, therefore, builds on previously published data against a wide range of viral pathogens [11], [39], [58], [61], [62], [63], [64]. 

## Summary

The results reported here expand our understanding of the comparative efficacy of a range of PVP-I formulations and other commonly used antiseptics. PVP-I demonstrated *in vitro* efficacy (>99.99% kill rate) against a range of bacterial and fungal pathogens with rapid onset of action, at high and low concentrations. By comparison, other antiseptics tested were generally effective *in vitro* at high concentrations, but efficacy was reduced on dilution. This is significant given the dilution of antiseptics during real-world use, for example, when washing or by wound exudate. Finally, PVP-I wound and oral products were also found to be more effective *in vitro* against CA16 and EV71, and had a faster onset of action than most other agents tested, suggesting that the use of PVP-I in infection control during HFMD outbreaks warrants further investigation.

It is important to note that this study had several limitations. A limited range of pathogens and antiseptics were assessed under clean conditions, *in vitro* efficacy against biofilms and the development of resistance were not evaluated. However, the *in vitro* efficacy of PVP-I against biofilms and a wider range of clinically relevant pathogens have been reported previously and the development of resistance has yet to be observed [17], [20], [21], [65]. Finally, as this study evaluated *in vitro* data only it is not possible to draw conclusions regarding clinical efficacy. Published trials and simulation studies have, however, demonstrated that PVP-I results in >99% reduction in bacterial or viral load after 30 seconds contact time in a number of settings (e.g., prevention of surgical site infections, antisepsis before nasotracheal intubation, mouth antisepsis for prevention of respiratory infections) [41], [66], [67], [68]. Therefore, the *in vitro* data presented in this study provides further support for the efficacy of PVP-I against a wide range of clinically relevant pathogens.

## Conclusions

All PVP-I products tested were highly efficacious *in vitro* (>99.99% kill rate) against a panel of bacteria and fungi relevant in wound, oral, vaginal and skin antisepsis, as well as against the HFMD enteroviruses CA16 and EV71. Furthermore, all PVP-I formulations tested demonstrated a rapid onset of action both when undiluted and at 1:10 dilution. This study provides valuable insights into the *in vitro* efficacy of a range of commonly used antiseptics, and may help to inform healthcare professionals to select appropriate antiseptics.

## Notes

### Competing interests

Eng Lee Tan and Nur Humaira Johari received service contracts from Mundipharma for the study and have done consultancy and speaker engagements for Mundipharma.

### Author contributions

Both authors (Eng Lee Tan and Nur Humaira Johari) contributed equally to study design and data analysis; Nur Humaira Johari conducted the experiments. Both authors drafted the initial manuscript and revised it critically for important intellectual content, approved the final manuscript as submitted, and agree to be accountable for all aspects of the work. 

### Funding information

This study was funded by Mundipharma Singapore Holding Pte. Limited. Medical writing support for the preparation of this article was provided by Dr. Jess Healy of Oxford PharmaGenesis, Oxford, UK, and was funded by Mundipharma GmbH, Basel, Switzerland. 

## Figures and Tables

**Table 1:**
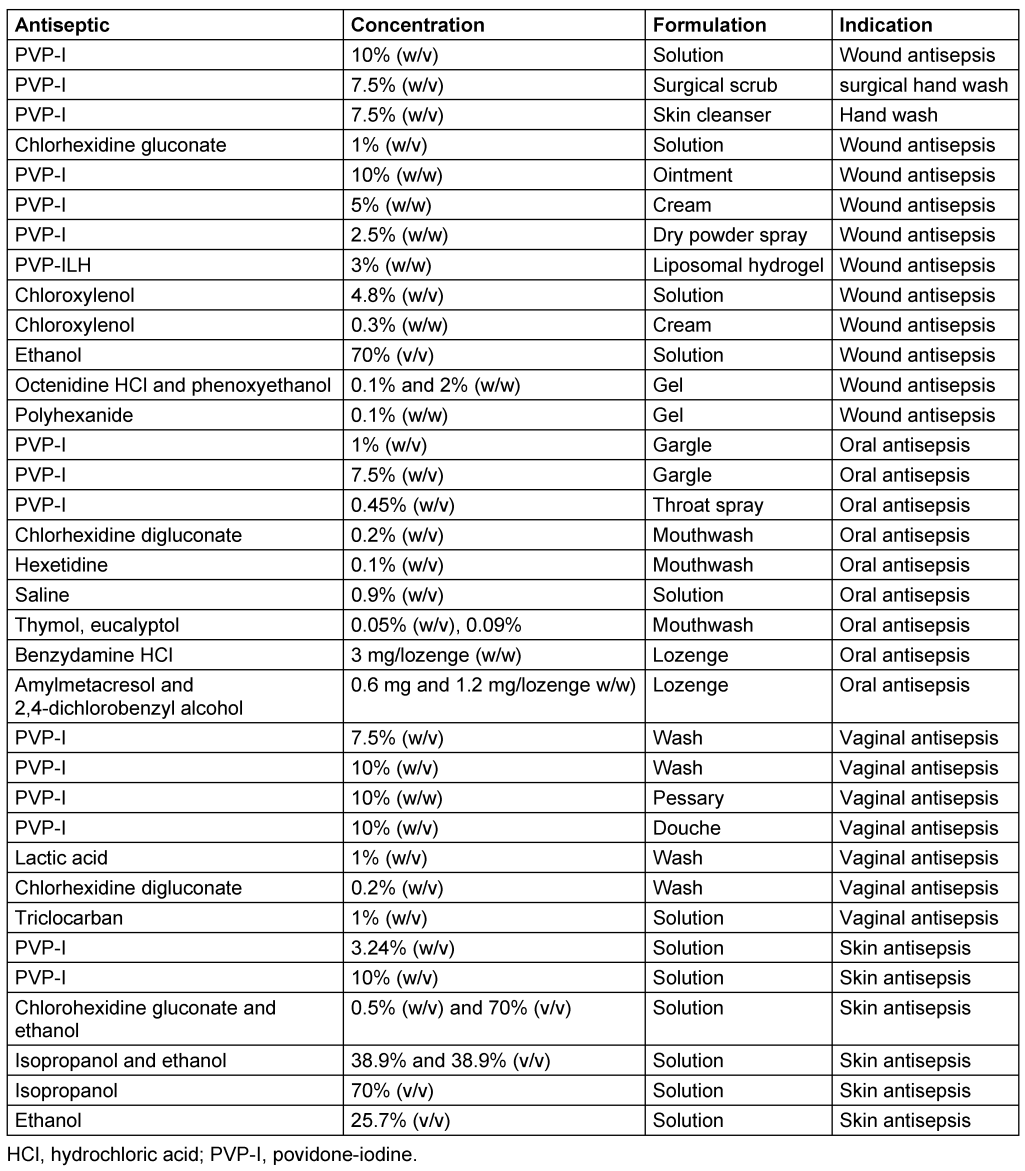
Tested antiseptic and antimicrobial products

**Table 2:**
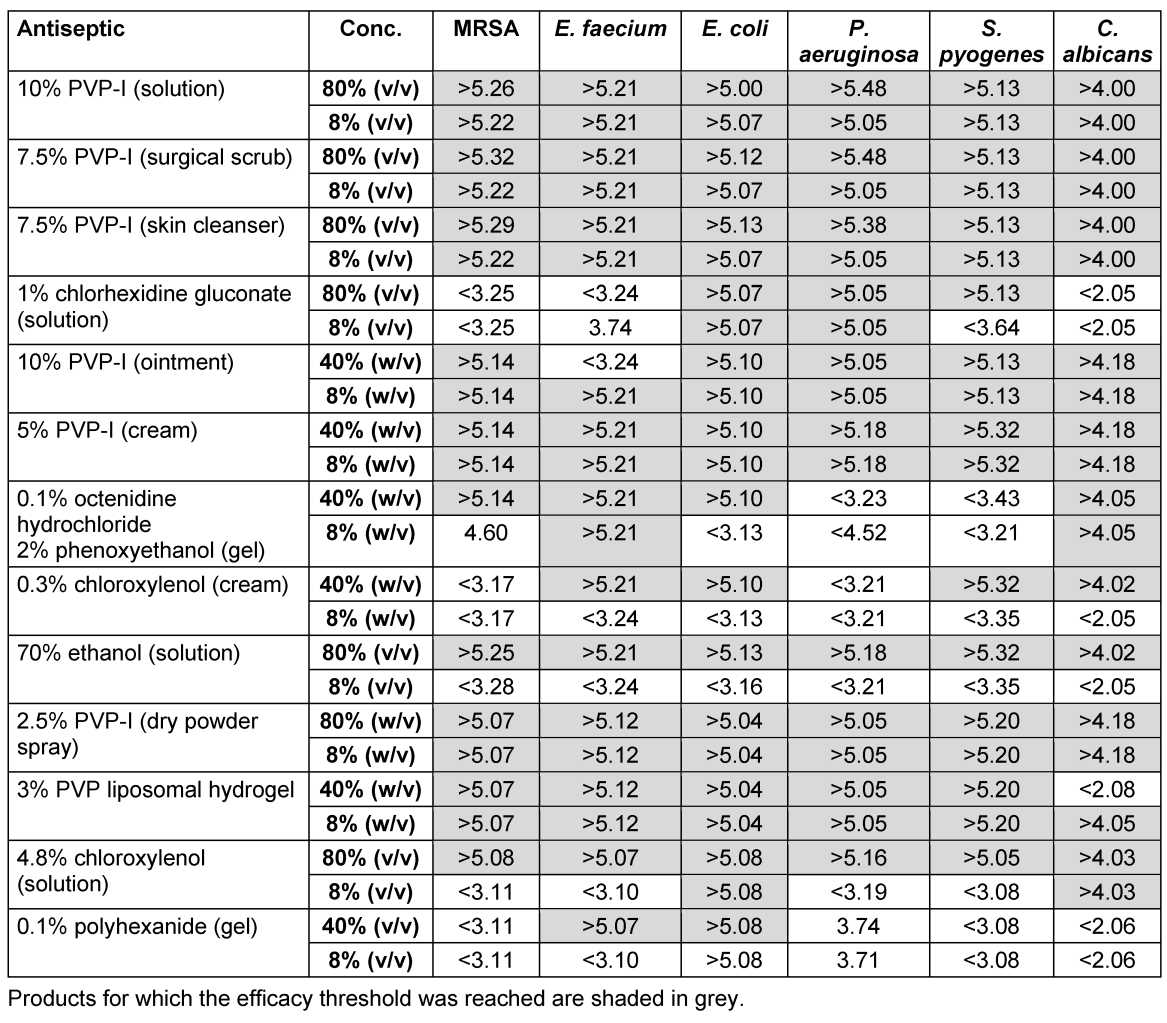
*In vitro* efficacy of selected wound antiseptics against six clinically relevant pathogens (lg reduction)

**Table 3:**
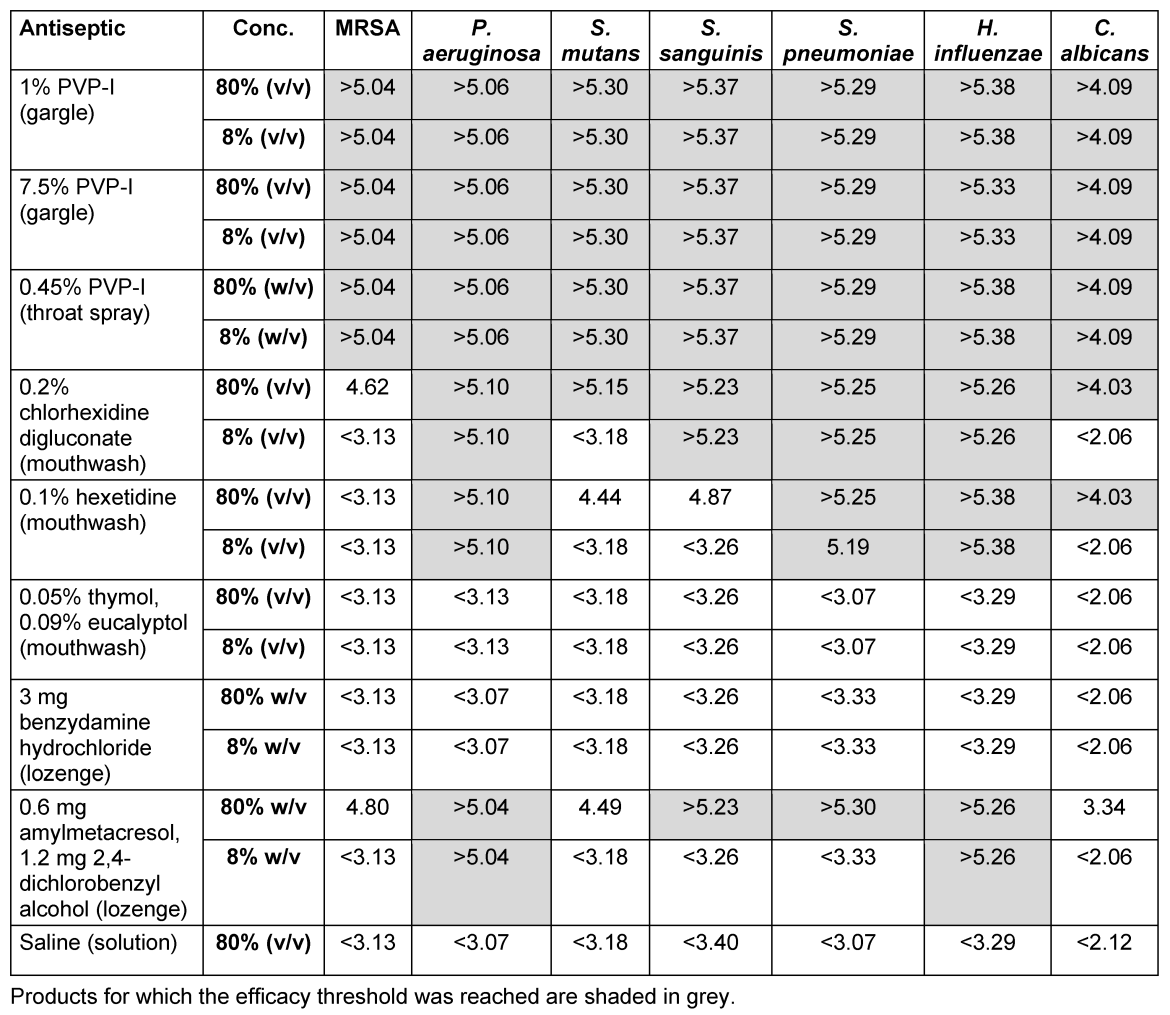
*In vitro* efficacy of selected oral antiseptics against seven clinically relevant pathogens (lg reduction)

**Table 4:**
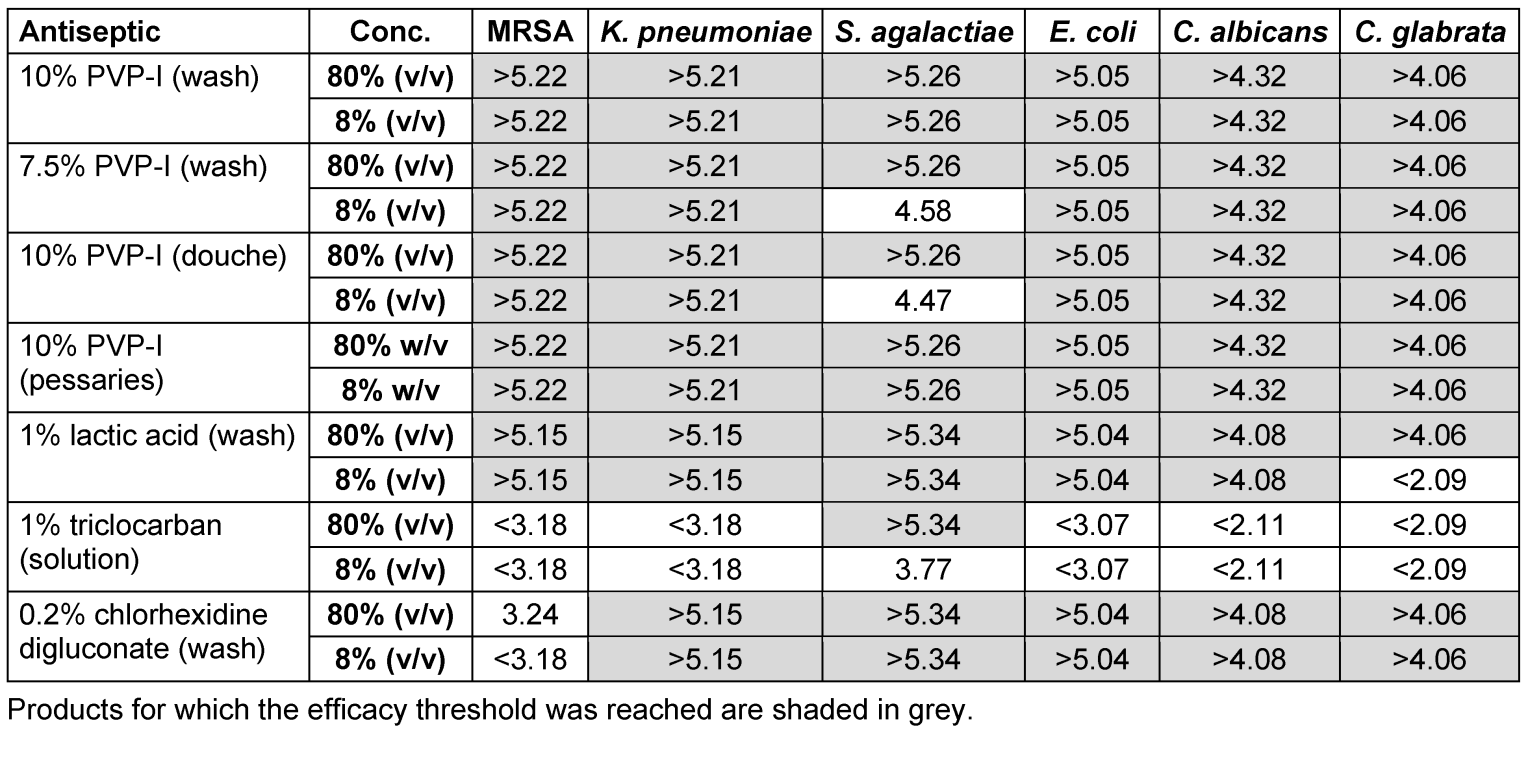
*In vitro* efficacy of selected feminine care antiseptics against six clinically relevant pathogens (lg reduction)

**Table 5:**
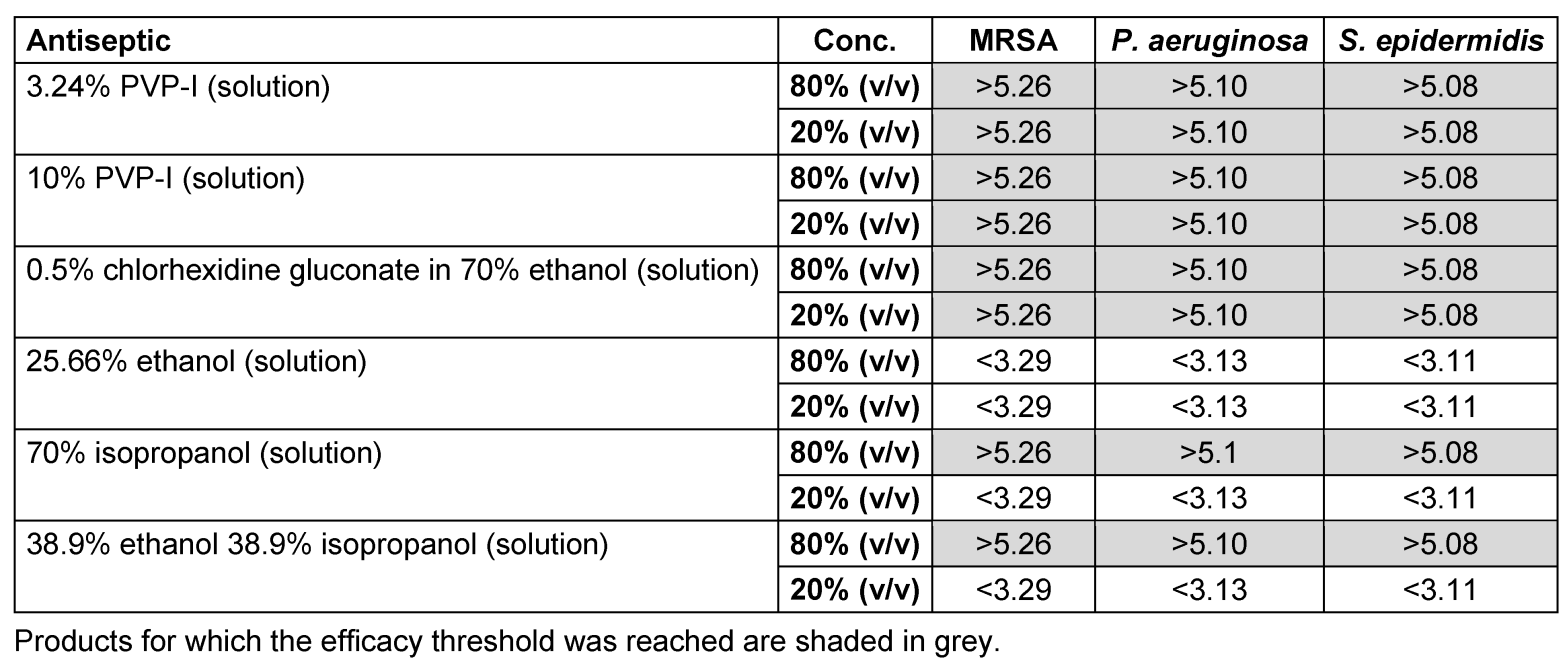
*In vitro* efficacy of selected skin care antiseptics against three clinically relevant pathogens (lg reduction)
